# Discovering the Potential Value of Coenzyme Q10 in Oxidative Stress: Enlightenment From a Synthesis of Clinical Evidence Based on Various Population

**DOI:** 10.3389/fphar.2022.936233

**Published:** 2022-07-14

**Authors:** Yili Zhang, Xinyi Huang, Ning Liu, Mengmin Liu, Chuanrui Sun, Baoyu Qi, Kai Sun, Xu Wei, Yong Ma, Liguo Zhu

**Affiliations:** ^1^ School of Traditional Chinese Medicine & School of Integrated Chinese and Western Medicine, Nanjing University of Chinese Medicine, Nanjing, China; ^2^ School of Traditional Chinese Medicine, Beijing University of Chinese Medicine, Beijing, China; ^3^ Wangjing Hospital, China Academy of Chinese Medical Sciences, Beijing, China

**Keywords:** oxidative stress, ferroptosis, evidence-based medicine, coenzyme Q10, mechanism

## Abstract

**Background:** Oxidative stress (OS) is associated with ferroptosis. Coenzyme Q10 (CoQ10), as an adjuvant treatment, has shown to be beneficial against OS. However, the efficacy of CoQ10 as a therapeutic agent against OS has not been promptly updated and systematically investigated.

**Methods:** A systematic literature search was performed using the Medline, EMBASE, Web of science, Cochrane Central Register of Controlled Trials, CNKI, CBM, Science direct and clinical trial. gov to identify randomized clinical trials evaluating the efficacy of CoQ10 supplementation on OS parameters. Standard mean differences and 95% confidence intervals were calculated for net changes in OS parameters using a random-effects model.

**Results:** Twenty-one randomized clinical studies met the eligibility criteria to be included in the meta-analysis. Overall, CoQ10 supplementation increased the levels of antioxidant enzymes [including superoxide dismutase (SOD) (SMD = 0.63; 95% CI: 0.38 to 0.88; *p* < 0.001), catalase (CAT) (SMD = 0.44; 95% CI:0.16 to 0.72; *p* = 0.002)] significantly and the levels of malondialdehyde (MDA) (SMD = -0.68; 95% CI: 0.93 to -0.43; *p* < 0.001) was decreased considerably. However, significant associations were not observed between this supplement and total antioxidant capacity (TAC), glutathione peroxidase (GPx) activity.

**Conclusion:** CoQ10 can improve OS as indicated by statistical significance in CAT and MDA concentrations, as well as SOD activity. Future studies focusing on long-term results and specific valuation of OS parameters are required to confirm the efficacy of CoQ10 on OS. We also believe that with the further research on ferroptosis, CoQ10 will gain more attention.

**Systematic Review Registration:** [https://inplasy.com/], identifier [INPLASY2021120123].

## Introduction

There are encouraging data showing that life expectancy has been increased for most of the past century. However, with aging, high rates of debilitating diseases become a major public health burden. Aging is a natural and complex physiological process influenced by many factors (Glatt et al., 2007; Flatt, 2012). Actually, one of the most accepted theories to explain aging is damage accumulation driven by oxidative stress (OS) (Finkel, Holbrook; Zhang et al., 2020; Marianna et al., 2020). Numerous studies have shown mitochondrial bioenergetic deterioration, particular with reactive oxygen species- (ROS-) induced mitochondrial DNA damage, is a prominent risk factor in normal physiological aging and in the pathogenesis of various cell and tissue (Nita and Grzybowski, 2016; Srivastava, 2017).

Coenzyme Q10 (CoQ10, ubiquinone), a redox-active lipid, it is mainly found in the inner mitochondrial membrane. All observations allowed the identification of CoQ10 as an obligate component in the electron transport chain and it is associated with the process of oxidative phosphorylation. Tissues with elevated energy demands, such as the heart, skeletal muscle and neurons, cultured with high concentrations of CoQ10. Moreover, the tissue concentration of CoQ10 declines with ageing and oxidative stress. All these observations demonstrated its bioenergetic role and this forms the basis of the clinical recommendation and application for CoQ10 (Lass et al., 1999; Díaz-Casado et al., 2019).

Starting from these premises, the supplementation with CoQ10 to maintain adequate tissue concentrations should be therapeutically promising. Nevertheless, the results obtained were contradictory. Additionally, weak search strategy, not registered in advance, monotonous types of diseases, and lack of assessing CoQ10 impact on comprehensive OS markers such as TAC are limitations in previous systematic reviews and meta-analysis (Jorat et al., 2019; Akbari et al., 2020; Alimohammadi et al., 2021). Hence, it gave us the opportunity to update the relevant evidence and conducted this study. We hope that the effects of CoQ10 supplementation on OS biomarkers can be comprehensively sort out and evaluated, as well as providing more accurate estimates of the overall effect to assess the future role of CoQ10 in the in the field of interest.

## Methods

This study was conducted and reported according to the guidelines of Preferred Reporting Items for Systematic Reviews and Meta-Analyses (PRISMA) checklist (Page et al., 2021). We registered the protocol of this study on the International Platform of Registered Systematic Review (Register number: INPLASY2021120123).

### Data Sources

Comprehensive computerized systematic searches were performed throughout PubMed/Medline, Web of science, Science direct, EMBASE, Cochrane Central Register of Controlled Trials, Chinese National Knowledge Infrastructure (CNKI), and Chinese Biomedical Literature Database (CBM) from inception until September 2021. In addition, we reviewed the reference lists of eligible reports, previous systematic reviews, guidelines, and contacted industry representatives to identify additional studies.

### Data Searches

The combination of MESH (Medical Subject Headings) and non-MESH terms were applied as follow (“Ubiquinone” OR “Coenzyme Q″) AND (“Antioxidants” OR “Reactive Oxygen Species” OR “Reactive Nitrogen Species” OR “Oxidative Stress” OR “Nitric Oxide” OR “Malondialdehyde” OR “Superoxide Dismutase” OR “Glutathione” OR “Glutathione Peroxidase” OR “Glutathione Reductase” OR “Oxidative Stresses” OR “stress oxidative” OR “GR” OR “Glutathione Lipoperoxidase” OR “GPx” OR “GSH-Px” OR “Glutathione” OR “SOD” OR “Malonyldialdehyde” OR “Malonylaldehyde” OR MDA OR “Nitrogen Monoxide” OR “Mononitrogen Mon-oxide” OR “NO” OR “Reactive Nitrogen Species” OR “Active Oxygen”) AND (“Random Allocation” OR “Single-Blind Method” OR “Double-Blind Method” OR “Cross-Over Studies” OR “Clinical Trials as Topic” OR RCT OR “Intervention Studies” OR “intervention” OR “controlled trial” OR “randomized” OR “randomized” OR “random” OR “randomly” OR “placebo” OR “assignment” OR “Cross-Over”). In addition, we manually searched relevant articles and clinical studies in order to find additional pertinent studies.

### Selection Criteria

All retrieved studies were evaluated independently by two reviewers (ZYL and HXY) according to the inclusion and exclusion criteria. Studies were included if they: 1) were randomized controlled trials with parallel design; 2) were performed on adult people (≥18 years old); 3) assessed the effects of oral coenzyme CoQ10 supplementation on aforementioned OS biomarkers compared with the placebo. No restrictions on the baseline health status, sex, race of the participants, and date.

Articles were excluded if 1) conducted on healthy people; 2) contained incomplete information about the selected outcomes; 3) surveyed the effects of medications containing CoQ10, where the specific effects of CoQ10 could not be discovered; 4) took CoQ10 for more than 6 months.

### Data Extraction

**FIGURE 1:**
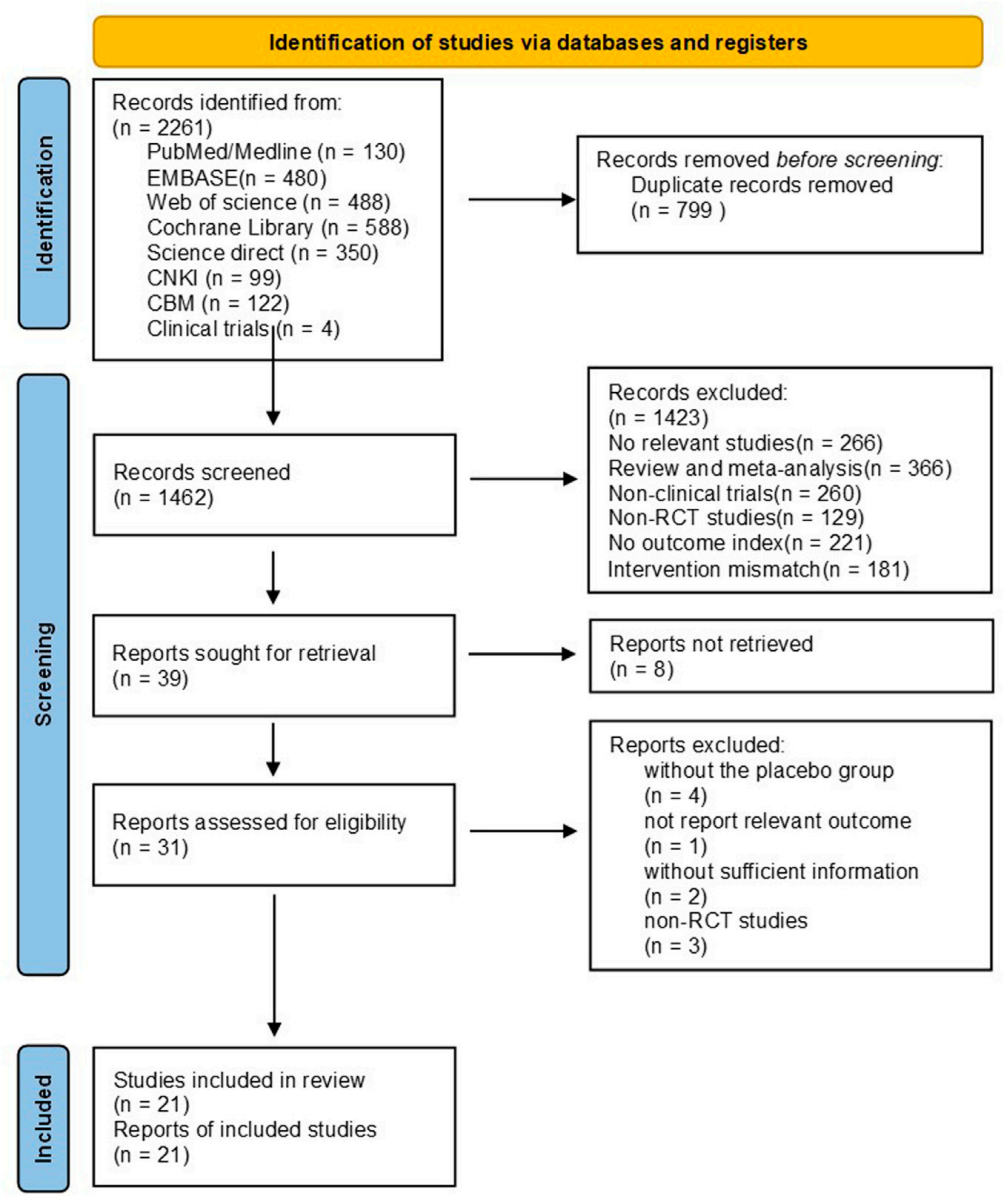
PRISMA flow chart displaying the search process and study selection.

Using a standardized pilot-tested form, paired reviewers screened titles and abstracts of identified citations, as well as full texts of potentially eligible studies (Figure 1). Multi-arm trials were included as a separate article in the meta-analysis. If any studies provided inadequate data for outcomes, we contacted the authors at least by email, and if we did not receive a response, we excluded the article from the analysis or recalculated data from the graphs using digital ruler software. Disagreements were resolved through discussion between the researchers or with an adjudicator’s help if there was a lack of consensus.

### Outcomes

MDA, SOD, TAC, NOx, GPx, and CAT were considered as the outcomes.

### Quality Assessment

The quality of the included RCTs was assessed independently by two reviewers according to the Cochrane Handbook for Systematic Review of Interventions, Version 5.1.0, including 1) sequence generation, 2) allocation concealment, 3) blinding of participants and personnel, 4) blinding of outcome assessment, 5) incomplete outcome data, 6) selective outcome reporting, and 7) other bias. “Low risk”, “high risk”, or “unclear risk” were the quality status.

### Data Synthesis

All analyses were performed using Stata version 16.0 (Stata Corp, College station, TX, United States). The overall effect sizes were assessed using the random-effects model (DerSimonian and Kacker, 2007). Standardized mean difference (SMD) was calculated by generic inverse variance. The heterogeneity was evaluated by using the Cochrane’s *Q*-test and the I^2^ index. I^2^ values < 25–50%, 50–75%, or >75% indicated mild, moderate, or severe heterogeneity, respectively.

### Subgroup Analysis and Sensitivity Analysis

Sub-group analysis was used to reduce the heterogeneity and sensitivity analysis was conducted to detect the impact of each study on the pooled effect size using the leave-one-out method (Sahebkar, 2014). The potential sources of heterogeneity were explored identified based on dosage, study duration, and types of diseases. Publication bias was tested by Egger’s test. Moreover, we used Engauge Digitizer version 10.8 to extract numerical estimates from graphs.

### Quality of the Evidence

The quality of the evidence would be assessed by the GRADE tool (Guyatt et al., 2008). Based on five key domains (methodology quality, directness of evidence, heterogeneity, precision of effect estimates and risk of publication bias), levels of evidence quality were defined as high, moderate, low and very low (Balshem et al., 2011).

## Results

### Study Selection and Characteristics

The initial search revealed 2,261 citations (130 from Medline, 480 from EMBASE, 488 from Web of science, 588 from Cochrane Central Register of Controlled Trials, 99 from CNKI, 122 from CBM, 350 from Science direct, and four from clinical trial. gov). After elimination of 799 duplicates, 1,462 records were retained for further evaluation. Articles were further screened by browsing their titles and abstracts, and the process was conducted by three independent authors (ZYL, HXY and LN). 1,423 articles were excluded as irrelevant to the topic, and a total of 39 articles remained for full-text review. 18 studies were excluded for the following reasons: studies did not retrieve (*n* = 8), studies did not report relevant outcomes (*n* = 1), trials without the placebo group (*n* = 4), trials without sufficient information (*n* = 2), and non-RCT studies (*n* = 3) (Figure 1).

The detailed characteristics of the 21studies are summarized in Table 1. Totally, 1,132 participants, including 577 cases and 555 controls, were participated in these studies. Published studies from 2000 to 2021, with 14 studies from Iran, four studies from China, two studies from Indonesia, and one study from India. Three trials exclusively included women or men. The mean age of participants varied between 19 and 76 years old.

**TABLE 1:** General characteristics of the included studies.

First author (year)	Country	Study design	Disease	Participants (EG/CG)	Gender (M/F)	Mean age (year)	Experimental group interventions	Control group interventions	Duration (week)	Outcome	Adverse events
Ram B. Singh Singh et al. (2009) 2000	India	Randomized, double-blind, placebo-controlled trial	Chronic renal failure	11/10	EG: 8/3	EG: 43.7 ± 10.2	180 mg/d	Placebo	4	MDA	NA
					CG: 7/3	CG: 44.2 ± 8.7					
A.Nadjarzadeh Nadjarzadeh et al. (2011) 2011	Iran	Randomized, double-blind, placebo-controlled trial	Idiopathic oligospermia	23/24	All men	EG: 34.17 ± 4.52	200 mg/d	Placebo	12	TAC、MDA	NA
						CG: 34.67 ± 6.69					
A. Nadjarzadeh Nadjarzadeh et al. (2014) 2012	Iran	Randomized, double-blind, placebo-controlled trial	Idiopathic oligospermia	23/24	All men	EG: 34.17 ± 4.52 CG: 34.67 ± 6.69	200 mg/d	placebo	12	CAT、SOD	NA
Bor-Jen Lee Lee et al. (2012) 2012	China	Randomized, placebo-controlled trial	CAD	16/12	EG: 14/2	EG: 73.0 ± 7.7	60 mg/d	Placebo	12	CAT、MDA	NA
					CG: 12/0	CG: 75.6 ± 7.9				SOD、GPx	
Bor-Jen Lee 2012	China	Randomized, placebo-controlle trial	CAD	15/12	EG: 14/1	EG: 77.1 ± 9.9	150 mg/d	Placebo	12	MDA、SOD	NA
					CG: 12/0	CG: 75.6 ± 7.9				GPx、CAT	
Bor-Jen Lee Lee et al. (2013) 2013	China	Randomized, double-blind, placebo-controlled trial	CAD	23/19	EG: 19/4	EG: 71.7 ± 11.5	300 mg/d	placebo	4	SOD、CAT	NA
					CG: 12/7	CG: 66.5 ± 11.1				GPx	
Meisam Sanoobar Sanoobar et al. (2013) 2013	Iran	Randomized, double-blind, placebo-controlled trial	Multiple sclerosis	22/23	EG: 2/20	EG: 33.1 ± 7.6	500 mg/d	Placebo	12	MDA、TAC	NA
					CG: 2/21	CG: 30.9 ± 7.7				SOD、GPx	
Mahdieh Abbasalizad Farhangi Farhangi et al. (2014) 2014	Iran	Randomized, double-blind, placebo-controlled trial	Nonalcoholic fatty liver disease	20/21	EG: 15/5	EG: 42.73 ± 10.77	100 mg/d	placebo	4	TAC 、MDA	NA
					CG: 16/5	CG: 42.18 ± 10.80					
Majid Mohammadshahi Mohammadshahi et al. (2014) 2014	Iran	Randomized, double-blind, placebo-controlled trial	Non-alcoholic fatty liver disease	20/21	NR	19–54 years	100 mg/d	placebo	12	MDA	NA
Mahsa Moazen Moazen et al. (2015) 2015	Iran	Randomised, single-blind, placebo-controlled trial	T2DM	26/26	EG: 16/10	EG: 50.67 ± 7.01	200 mg/d	placebo	8	MDA	NA
					CG: 12/14	CG: 52.79 ± 7.66					
Hadi Abdollahzad Abdollahzad et al. (2015) 2015	Iran	Randomized, double-blind, placebo-controlled trial	RA	22/22	EG: 3/19	EG: 48.77 ± 11.58	100 mg/d	placebo	8	MDA、TAC	NA
					CG: 2/20	CG: 50.41 ± 11.28					
M.J. Hosseinzadeh-Attar Hosseinzadeh-Attar et al. (2015) 2015	Iran	Randomized, double-blind, placebo-controlled trial	T2DM	31/33	EG: 19/12	EG: 45.2 ± 7.6	200 mg/d	placebo	12	NOx	NA
					CG: 18/15	CG: 47.1 ± 8.3					
Parvin Zarei Zarei et al. (2018) 2018	Iran	Randomized, double-blind, placebo-controlled trial	T2DM	34/34	All women	EG: 53.1 ± 6.23	100 mg/d	placebo	12	CAT、TAC	NA
						CG: 53.35 ± 6.56					
Mahtab Ramezani Ramezani et al. (2020) 2018	Iran	Randomized, double-blind, placebo-controlled trial	Ischemic stroke	21/23	EG: 9/12	EG: 64.10 ± 11.04	300 mg/d	placebo	4	SOD、MDA	NA
					CG: 13/10	CG: 62.04 ± 13.32					
Leila Jahangard Jahangard et al. (2019) 2019	Iran	Randomized, double-blind, placebo-controlled trial	Bipolar disorder	36/33	EG: 8/28	EG: 37.47 ± 10.69	100 mg/d	placebo	8	MDA、TAC	NA
					CG: 3/30	CG: 39.52 ± 10.82				CAT、NO	
Melika Fallah Fallah et al. (2019) 2019	Iran	Randomized, double-blind, placebo-controlled trial	Diabetic hemodialysis	30/30	NR	EG: 64.8 ± 11.5	120 mg/d	placebo	12	TAC、GSH	NA
						CG: 59.4 ± 12.2				MDA、NO	
Seyyede-Nadia Hosseini-Ghalibaf Hosseini-Ghalibaf et al. (2020) 2020	Iran	Randomized, double-blind, placebo-controlled trial	Bipolar Disorder	36/33	EG: 8/28	EG: 37.47 ± 10.69	200 mg/d	placebo	8	Saliva TAC	NA
					CG: 3/30	CG: 39.52 ± 10.82					
Seyyede-Nadia Hosseini-Ghalibaf et al. (2020)	Iran	Randomized, double-blind, placebo-controlled trial	Bipolar Disorder	36/33	EG: 8/28	EG: 37.47 ± 10.69	200 mg/d	placebo	8	Urine TAC	NA
					CG: 3/30	CG: 39.52 ± 10.82					
Keda Zhu Zhu et al. (2020) 2020	China	Randomized, double-blind, placebo-controlled trial	RA	45/45	EG: 10/35	EG: 48.15 ± 11.68	30 mg/d	placebo	12	TAC 、MDA	NA
					CG: 8/37	CG: 47.36 ± 12.11					
Carissa Adriana Adriana et al. (2021) 2021	Indonesia	Randomized, double-blind, placebo-controlled trial	Acne Vulgaris	18/18	NR	EG: 25.9 ± 4.52	100 mg/d	placebo	8	SOD	NA
						CG: 26.5 ± 5.82					
Maria Leleury Leleury et al. (2020) 2021	Indonesia	Randomized, double-blind, placebo-controlled trial	Acne Vulgaris	18/18	NR	EG: 25.9 ± 4.52	100 mg/d	placebo	8	GSH-Px	NA
						CG: 26.5 ± 5.82					
Yanhua Zhang Zhang et al. (2019) 2021	China	Randomized, double-blind, placebo-controlled trial	PCOS	67/66	NR	NR	200 mg/d	placebo	8	MDA	NA
Mohammad Amin Valizade Hasanloei Hasanloei et al. (2021) 2021	Iran	Randomized, double-blind, placebo-controlled trial	Traumatic mechanical ventilated patients	20/20	EG: 10/10	EG: 52.47 ± 7.26	400 mg/d	placebo	1	MDA	NA
					CG: 13/7	CG: 50.12 ± 9.66					

Note: TAC: EG: experiment group; CG: control group; total antioxidant capacity; CAT: catalase; CAD: coronary artery disease; RA: rheumatoid arthritis; T2DM: type 2 diabetes mellitus; PCOS: polycystic ovarian syndrome.

### Risk of Bias in Individual Studies

**FIGURE 2:**
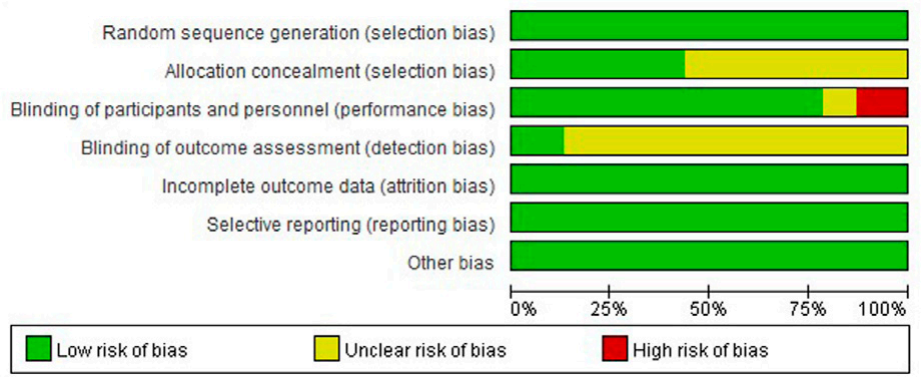
Risk of bias graph: review authors’ judgements about each risk of bias item presented as percentages across all included studies.

We used Cochrane risk of bias (ROB) tool for all included studies (Figure 2). There were three studies with high risk of performance bias, two studies with unclear risk of performance bias. There was unclear risk of selection bias in 13 studies, unclear risk of detection bias in 20 studies.

### Primary Results

#### MDA

**FIGURE 3:**
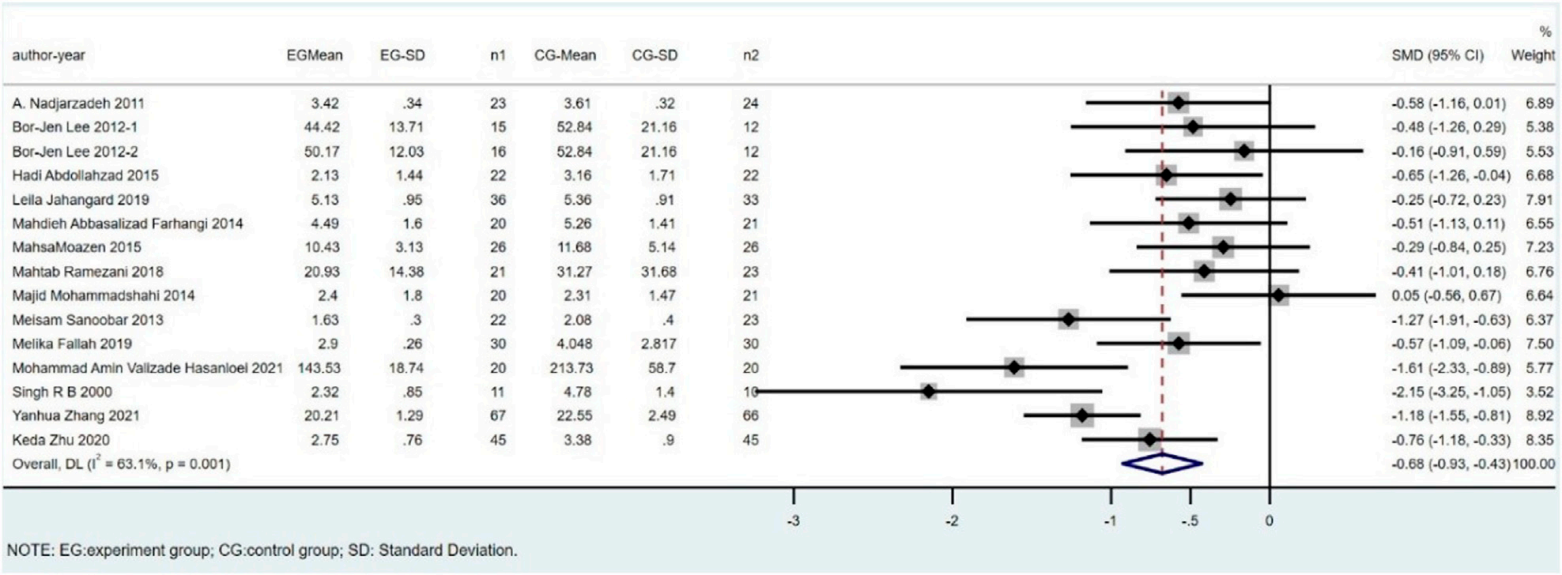
Meta-analysis of studies investigating the effect of CoQ10 supplements use on MDA level.

Fifteen studies, including 782 subjects, reported the outcome of MDA. The difference in MDA between the CoQ10 groups and placebo groups was significant (SMD = -0.68; 95% CI: -0.93 to -0.43; *p* < 0.001; I^2^ = 63.1%). (Figure 3).

### SOD

**FIGURE 4:**
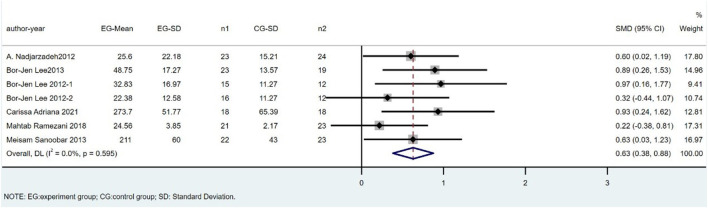
Meta-analysis of studies investigating the effect of CoQ10 supplements use on SOD level.

There were seven trials involving 269 individuals (138 cases and 131 controls) that compared SOD levels between CoQ10 and placebo groups. The difference in SOD between the CoQ10 groups and placebo groups was significant as shown in Figure 4 (SMD = 0.63; 95% CI: 0.38 to 0.88; *p* < 0.001), with no heterogeneity between studies (I^2^ = 0%; *p* = 0.60).

### TAC

**FIGURE 5:**
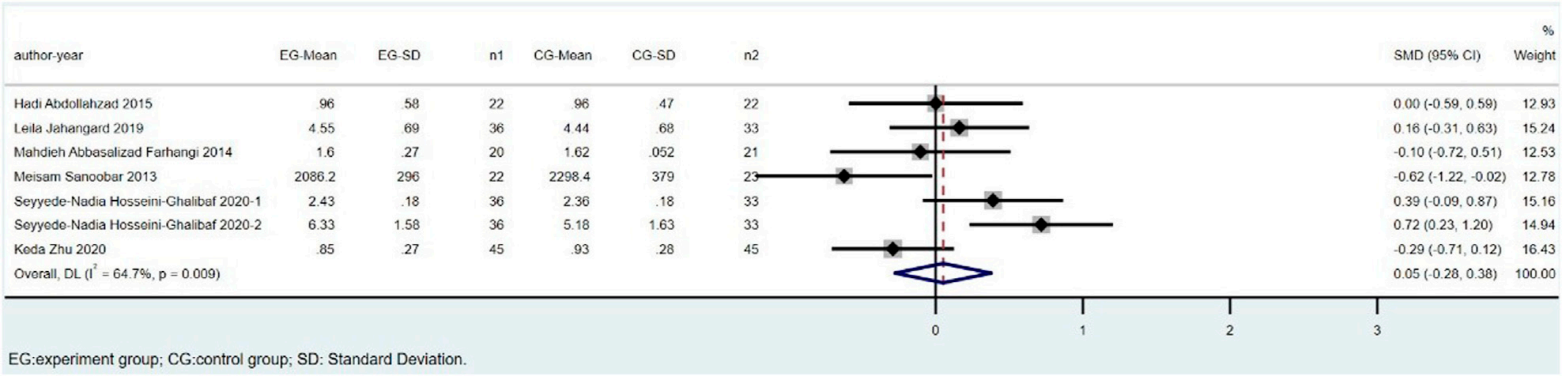
Meta-analysis of studies investigating the effect of CoQ10 supplements use on TAC level.

There were eight trials involving 495 individuals (251 cases and 244 controls) that compared TAC levels between CoQ10 and placebo groups. Parvin Zarei’s study was excluded because of the high heterogeneity. The overall estimates showed that TAC levels did not significantly differ between the CoQ10 and placebo groups (SMD = 0.05; 95% CI: -0.28 to 0.38; *p* = 0.764), with a high heterogeneity between studies (I^2^ = 64.7%; *p* = 0.009) (Figure 5).

### GPx

**FIGURE 6:**
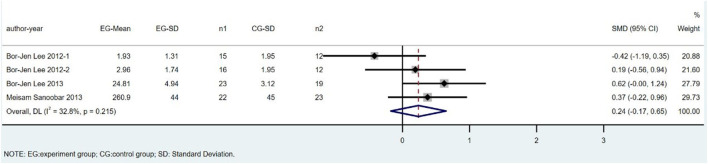
Meta-analysis of studies investigating the effect of CoQ10 supplements use on GPx level.

There were four trials involving 142 individuals (76 cases and 66 controls) that compared GPx levels between CoQ10 and placebo groups. The overall estimates showed that GPx levels did not significantly differ between the CoQ10 and placebo groups (SMD = 0.24; 95% CI: 0.17 to 0.65; *p* = 0.26), with a low heterogeneity between studies (I^2^ = 32.8%; *p* = 0.22) (Figure 6).

**FIGURE 7:**
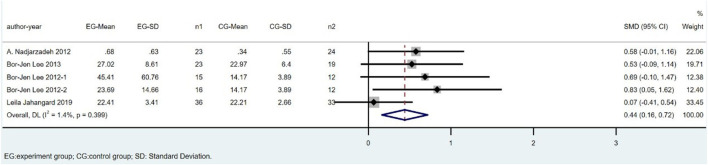
Meta-analysis of studies investigating the effect of CoQ10 supplements use on CAT level.

Six studies, including 281 subjects, reported the outcome of CAT. We excluded Parvin Zarei’s study because of the high heterogeneity. The difference in CAT between the CoQ10 groups and placebo groups was significant (SMD = 0.44; 95% CI: 0.16 to 0.72; *p* = 0.002), with low heterogeneity between studies (I^2^ = 1.4%; *p* = 0.399) (Figure 7).

### Other Indicators

#### NOx

Three studies, including 193 subjects, reported the outcome of NOx. The difference in NOx between the CoQ10 groups and placebo groups was significant (SMD = -0.59; 95% CI: -0.88 to -0.30; *p* < 0.001), with no heterogeneity between studies (I^2^ = 0%; *p* = 0.66).

### Subgroup Analysis

A subgroup meta-analysis comparing studies with different dosages, duration, and types of diseases was conducted. Patients were investigated at six different kinds of diseases: cardiology (*n* = 3), rheumatoid arthritis (*n* = 2), bipolar disorder (*n* = 1), nonalcoholic fatty liver disease (*n* = 2), diabetes mellitus type 2 (*n* = 2) and other diseases (*n* = 5). The results suggested that different diseases may be responsible for heterogeneity among MDA, TAC, CAT, and GPx. The detailed results for subgroup analyses are summarized in Supplementary Table S1; Supplementary Figures S1–S4.

### Sensitivity Analysis and Publication Bias

Sensitivity analysis indicated that the exclusion of any primary studies did not influence the pooled estimates other than GPx (Supplementary Figures S5–S8).

The Egger test for MDA index showed no evidence of publication bias (*t* = -0.11, *p* = 0.918).

### GRADE Assessment

We used the GRADE assessment to evaluate the evidence regarding the effect of CoQ10 supplementations compared with placebo (Supplementary Table S2). According to this evaluation, there was evidence that CoQ10 supplementation decreased the level of MDA in various population, as well as increased the level of SOD. Although the supplementary CoQ10 had a low impact on all indicators, this was due to the inconsistencies and indirectness of the results.

## Discussion

### The Main Findings and Strength of This Study

The study illustrated that CoQ10 supplementation can directly or indirectly through increasing SOD and CAT levels significantly, decreasing the levels of MDA considerably and may thereby affect OS and subsequent pathophysiological events. However, significant associations were not observed between this supplement and TAC, GPx activity, which differs from some previous studies (Alimohammadi et al., 2012; Akbari et al., 2020; Sangsefidi et al., 2020) and may be attributed to the generalizability of the included population and the diversity of disease types. Additionally, we also conducted subgroup meta-analysis comparing studies with different dosages, duration, and types of diseases. The findings revealed the value of CoQ10 in different scope of application more accurately. Also, the differences in study population’s characteristics, the dosage of CoQ10 used and the duration of intervention might explain the discrepancies to a certain extent among current evidence.

To our best knowledge, although our meta-analysis is not the first study investigating the effect of CoQ10 on OS biomarkers in human, we believed that the application of robust search strategy and study design, a wide variety of population (disease) as well as the transparency of the entire study process (the protocol registration has been completed on INSPLASY) were among the strengths of this study.

Of note, in order to better guide clinical practice, further evaluation of the evidence was carried out by the GRADE approach. Due to the moderate and high quality of included trials and the above outcomes from individual trials, we are confident that the diagnosis and treatment of oxidative stress in the future will be addressed in the guidelines.

### Coenzyme Q10 Affects Antioxidant Enzyme Activity

In the systematic review and meta-analysis, we confirmed that the antioxidant capacity of CoQ10 has an important role in reducing the production of free radicals, which can ultimately lead to a reduction in MDA levels. Furthermore, antioxidant enzymes such as SOD and CAT are responsible for neutralizing the free radicals. The activity of these enzymes increases in body followed by the consumption of antioxidants, such as CoQ10.

MDA is the best product to evaluate lipid peroxidation (Sepidarkish et al., 2020), and it has been shown that CoQ10 can prevent lipid peroxidation and reduce MDA *in vitro* and *in vitro* (Talevi et al., 2013; Gholnari et al., 2018; Hormozi et al., 2019). CoQ10 supplementation may reduce MDA levels via several mechanisms. Firstly, CoQ10, as a part of the mitochondrial respiratory chain, inhibits mitochondrial endogenous ROS production (Jing et al., 2015), and lower ROS is associated with reduced MDA levels (Karajibani et al., 2009). Besides, lipid metabolism was regulated by CoQ10, which can prevent lipid oxidation in a variety of cellular and gene expression ways.

The results of our study confirmed that CoQ10 significantly increases SOD activity. SOD is known to be one of the main detoxifying enzymes in the mitochondria (Zou et al., 2017). One of the possible mechanisms is that CoQ10, as a mitochondrial antioxidant, works by decreasing ROS in the mitochondria, so increasing the activity of SOD (Sourris et al., 2012). Besides, CoQ10 is also able to promote the expression of FOXO3a, which has been shown to increase SOD gene expression (Abd El-Aal et al., 2017).

The results of our meta-analysis also indicated that CoQ10 supplementation increased CAT activity in a non-significant manner. In addition, the results showed that CoQ10 supplementation had no significant effect on NO levels. Primary studies of high heterogenicity and low numbers maybe the reasons that we could not find a significant effect of CoQ10 supplementation on these indicators. However, the results of other studies investigating the effect of CoQ10 supplementation on NO concentrations are also controversial (Erol et al., 2010), and there are no concrete conclusions in these regards.

### The Rise of Ferroptosis Concept and Potential Relationship With OS and CoQ10

Cell death has an important role in the process of development, homeostasis, and pathogenesis of acute and chronic diseases (Van Opdenbosch and Lamkanfi, 2019). Ferroptosis is a new kind of regulated cell death involving various biology processes, such as iron metabolism, lipid metabolism, OS and CoQ10 (Qiu et al., 2020).

Iron, a potentially toxic molecule, can catalyze reactive oxygen species (ROS) radical formation through the Fenton reaction, in which H2O2 is reduced by a single electron to produce hydroxyl radicals (Bresgen and Eckl, 2015); this ultimately leads to damage to various cellular structures.

OS is a common pathogenesis of many chronic diseases. Iron metabolism could be the basis for the dynamic interplay between oxidative stress and antioxidants in many pathophysiological processes. The redox state is affected by iron deficiency and iron overload, which can be restored using iron supplementation and iron chelation, respectively. Likewise, antioxidants combining with basis treatment has been suggested for attenuating tissue damage caused by oxidative stress.

Therefore, antioxidants could be useful for protecting cells and tissues as well as reversing oxidative damage. CoQ10 is an endogenous antioxidant produced by the mevalonate pathway, which is important component of the mitochondrial respiratory chain. Multiple studies have shown that the regulatory role of CoQ10 in uncoupling proteins’ activation (Parikh et al., 2009), cell signaling (Groneberg et al., 2005), cell growth (Crane, 1999), and cell death (Alleva et al., 2001). In recent research of ferroptosis with CoQ10, ferroptosis suppressor protein 1 (FSP1) has been recognized as an oxidoreductase of CoQ10 to reduce it at the plasma membrane and can strengthen the resistance of cells to ferroptosis (Bersuker et al., 2019). Notably, CoQ10 deficiency currently is the only treatable mitochondrial disorder, however, little is known about how it may affect other organelles. High concentrations of CoQ10 have been found on lysosomal membranes, which has been proposed to be associated with normal acidification of the lysosomal lumen (Heaton et al., 2020). Additionally, Rizzardi N et al. in a study of cultured cells demonstrated that, protecting of membrane lipids from peroxidation and increasing cellular resistance to ferroptotic stimuli can be achieved by supplementation with exogenous CoQ10 (Rizzardi et al., 2021).

Greater interest has been shown in improving our understanding of the relationship between ferroptosis and various diseases, but there are few studies on the downstream effects of ferroptosis; that is, how it triggers disease occurrence and progression. Hence, it is vital to elucidate the mechanisms of ferroptosis in different diseases. Future studies are needed to investigate the basis of ferroptosis and to identify relationship between ferroptosis and other forms of cell death in dynamic environments. This would improve our understanding of the occurrence, progression, diagnosis, and treatment of ferroptosis-related disorders. We believe that it will provide new ideas for the treatment of ferrroptosis-related diseases via focuses on the relationship between antioxidant systems and ferroptosis and reveals the inhibitory role of antioxidant system in ferroptosis.

### Future Direction and the Limitations of This Study

There are multiple studies combining CoQ10 with other drugs, making it difficult to assess CoQ10 specific effect. Another limitation is the poor oral bioavailability of CoQ10. However, due to the relatively easy availability and high safety profile of CoQ10, more randomized studies are required to assess its’ role in various diseases. The development of inhalator CoQ10 is a promising approach and may prove useful in the future.

It is vital to acknowledge some limitations of our study. Firstly, information on the formulation of CoQ10 supplementation used in clinical trials was not available, and different pharmacokinetic properties may affect the bioavailability of various formulations, thus affecting the effect of CoQ10. Secondly, the heterogeneity within the studied factors may be due to various diseases, study durations (1–12 weeks), supplemental doses (30–500 mg/day), patients’ initial antioxidant serum levels, and patients’ other characteristics, such as gender and age. Moreover, the clinical trials included in this meta-analysis had limited sample sizes and follow-up periods.

## Conclusion

We concluded that CoQ10 can improve OS by statistical significance in CAT and MDA concentrations, as well as SOD activity, compared with placebo. Future studies should confirm its efficacy of CoQ10 on OS by assessing the long-term results and specific evaluation of OS parameters. With the increasing maturity of ferroptosis phenotype in the pathogenesis of various diseases, we believe that CoQ10 will have more promising scientific and clinical applications in the future.

## Data Availability

The original contributions presented in the study are included in the article/Supplementary Material, further inquiries can be directed to the corresponding authors.
