# Supplementary material for: Discovering the Potential Value of Coenzyme Q10 in Oxidative Stress: Enlightenment From a Synthesis of Clinical Evidence Based on Various Population
Source: Front Pharmacol. 2022 Jul 14;13:936233. doi: 10.3389/fphar.2022.936233 (PMC9330130; doi:10.3389/fphar.2022.936233)
Supplement: Supplementary file 1 [file DataSheet1.docx]

**Subgroup analysis**

Supplementary Table 1: Meta-analysis presenting the effects of CoQ10 on indicators of oxidative stress based on different subgroups.

| Indicators | Study group | Number of trials | Meta-analysis  SMD (95 % CI) | P value | Heterogeneity  I2 (%) |
| --- | --- | --- | --- | --- | --- |
| MDA |  |  |  |  |  |
|  | **Dosage (mg/d)** |  |  |  |  |
|  | ≤100 | 7 | -0.868 (-1.642, -0.093) | 0.028 | 91.7 |
|  | (100, 200] | 7 | -1.224 (-1.930, -0.517) | 0.001 | 89.4 |
|  | ＞200 | 3 | -1.078 (-1.788, -0.368) | 0.003 | 71.9 |
|  | **Study duration (week)** |  |  |  |  |
|  | ≤4 | 4 | -1.087(-1.837, -0.336) | 0.005 | 76.6 |
|  | 8 | 5 | -1.170(-2.066, -0.273) | 0.011 | 92.9 |
|  | 12 | 8 | -0.948(-1.642, -0.254) | 0.007 | 90.2 |
|  | **Subject disease** |  |  |  |  |
|  | Cardiovascular | 3 | -0.361(-0.761, 0.039) | 0.077 | 0.0 |
|  | RA | 2 | -0.722(-1.071, -0.372) | ≤0.001 | 0.0 |
|  | Bipolar disorder | 1 | -0.247(-0.721, 0.227) | 0.307 | - |
|  | Nonalcoholic fatty liver disease | 2 | -0.225(-0.780, 0.330) | 0.426 | 38.1 |
|  | T2DM | 2 | -0.442(-0.817, -0.066) | 0.021 | 0.0 |
|  | Others | 5 | -1.243(-1.653, -0.834) | ≤0.001 | 53.4 |
| TAC |  |  |  |  |  |
|  | **Dosage (mg/d)** |  |  |  |  |
|  | ≤100 | 5 | 0.545(-0.466, 1.556) | 0.291 | 94.3 |
|  | (100, 200] | 2 | 0.549(0.208, 0.890) | 0.002 | 0.0 |
|  | ＞200 | 1 | -0.622(-1.221, -0.023) | 0.042 | - |
|  | **Study duration (week)** |  |  |  |  |
|  | ≤4 | 1 | -0.104(-0.717, 0.509) | 0.739 | - |
|  | 8 | 4 | 0.336(0.040, 0.632) | 0.026 | 27.9 |
|  | 12 | 3 | 0.704(-1.300, 2.708) | 0.491 | 97.4 |
|  | **Subject disease** |  |  |  |  |
|  | RA | 2 | -0.195(-0.535, 0.145) | 0.262 | 0.0 |
|  | Bipolar disorder | 3 | 0.418(0.103, 0.733) | 0.009 | 22.9 |
|  | Nonalcoholic fatty liver disease | 1 | -0.104(-0.717, 0.509) | 0.739 | - |
|  | T2DM | 1 | 3.068(2.361, 3.775) | ≤0.001 | - |
|  | Others | 1 | -0.622(-1.221, -0.023) | 0.042 | - |
| GPx |  |  |  |  |  |
|  | **Dosage (mg/d)** |  |  |  |  |
|  | ≤100 | 1 | 0.191(-0.559, 0.941) | 0.618 | - |
|  | (100, 200] | 1 | -0.419(-1.187, 0.349) | 0.285 | - |
|  | ＞200 | 2 | 0.490(0.062, 0.918) | 0.025 | 0.0 |
|  | **Study duration (week)** |  |  |  |  |
|  | ≤4 | 1 | 0.621(-0.002, 1.244) | 0.051 | - |
|  | 12 | 3 | 0.094(-0.365, 0.554) | 0.688 | 24.0 |
|  | **Subject disease** |  |  |  |  |
|  | Cardiovascular | 3 | 0.163(-0.435, 0.762) | 0.593 | 53.0 |
|  | Others | 1 | 0.373(-0.217, 0.963) | 0.215 | - |
| CAT |  |  |  |  |  |
|  | **Dosage (mg/d)** |  |  |  |  |
|  | ≤100 | 3 | 2.615(-0.588, 5.817) | 0.110 | 98.0 |
|  | (100, 200] | 2 | 0.615(0.147, 1.083) | 0.010 | 0.0 |
|  | ＞200 | 1 | 0.526(-0.092, 1.145) | 0.095 | - |
|  | **Study duration (week)** |  |  |  |  |
|  | ≤4 | 1 | 0.526(-0.092, 1.145) | 0.095 | - |
|  | 8 | 1 | 0.065(-0.407, 0.538) | 0.787 | - |
|  | 12 | 4 | 2.232(0.078, 4.386) | 0.042 | 96.5 |
|  | **Subject disease** |  |  |  |  |
|  | Cardiovascular | 3 | 0.656(0.244, 1.068) | 0.002 | 0.0 |
|  | Bipolar disorder | 1 | 0.065(-0.407, 0.538) | 0.787 | - |
|  | T2DM | 1 | 7.134(5.828, 8.441) | ≤0.001 | - |
|  | Others | 1 | 0.576(-0.008, 1.160) | 0.053 | - |

Supplementary Table 2: GRADE evidence for randomized controlled trials.

| Outcomes | No of Participants  (studies) | Quality of the evidence (GRADE) | Risk difference with CoQ10 (95% CI) |
| --- | --- | --- | --- |
| MDA | 782 (15 studies) | ⊕⊕⊝⊝ LOW due to inconsistency due to indirectness | The mean MDA in the intervention groups was 0.66 standard deviations lower (0.91 to 0.42 lower) |
| SOD | 269 (7 studies) | ⊕⊕⊝⊝ LOW due to inconsistency due to indirectness | The mean SOD in the intervention groups was 0.62 standard deviations higher (0.37 to 0.86 higher) |
| TAC | 495 (8 studies) | ⊕⊕⊝⊝ LOW due to inconsistency due to indirectness | The mean TAC in the intervention groups was 0.39 standard deviations higher (0.24 lower to 1.02 higher) |
| GPx | 142 (4 studies) | ⊕⊕⊝⊝ LOW due to inconsistency due to indirectness | The mean GPx in the intervention groups was 0.24 standard deviations higher (0.17 lower to 0.64 higher) |
| CAT | 281 (6 studies) | ⊕⊕⊝⊝ LOW due to inconsistency due to indirectness | The mean CAT in the intervention groups was 1.49 standard deviations higher (0.26 to 2.73 higher) |

Note: MDA: Malondialdehyde; SOD: Superoxide dismutase; TAC: total antioxidant capacity; GPX: glutathione peroxidase; CAT: Catalase.

**MDA**


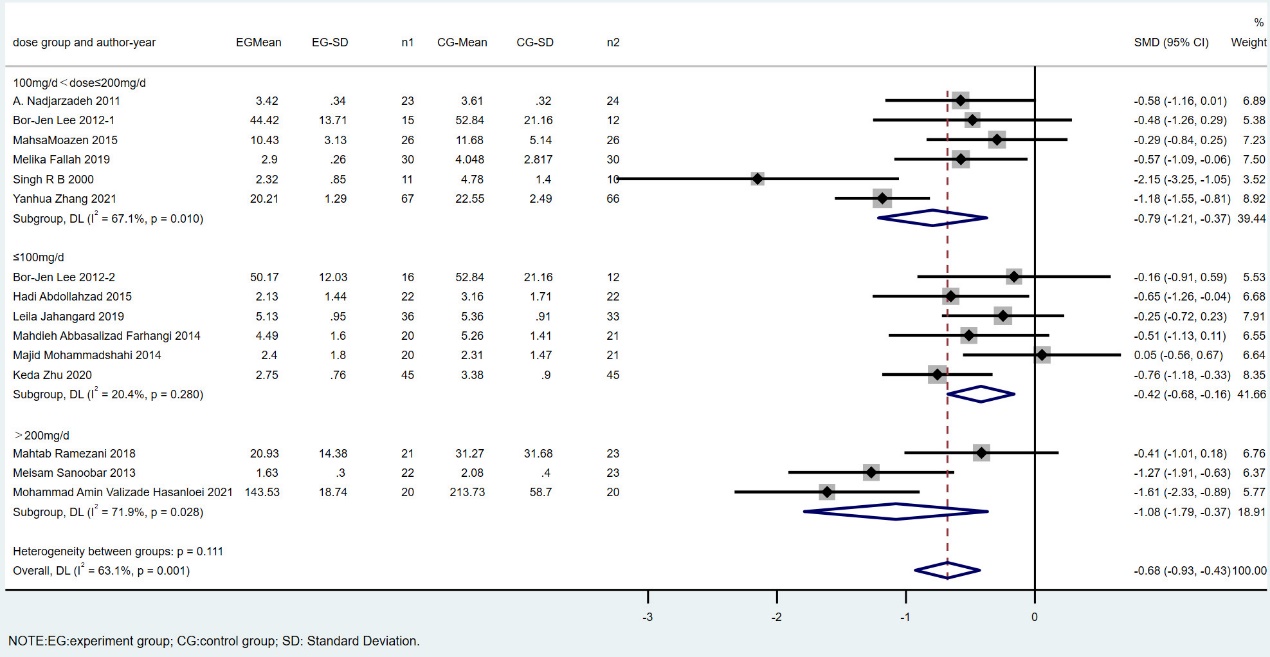


Supplementary Figure 1a: Meta-analysis presenting the effects of CoQ10 on MDA based on different dosages subgroups. SMD: Stand Mean Difference; CI: Confidence Interval; EG: experiment group; CG: control group; SD: Standard Deviation.


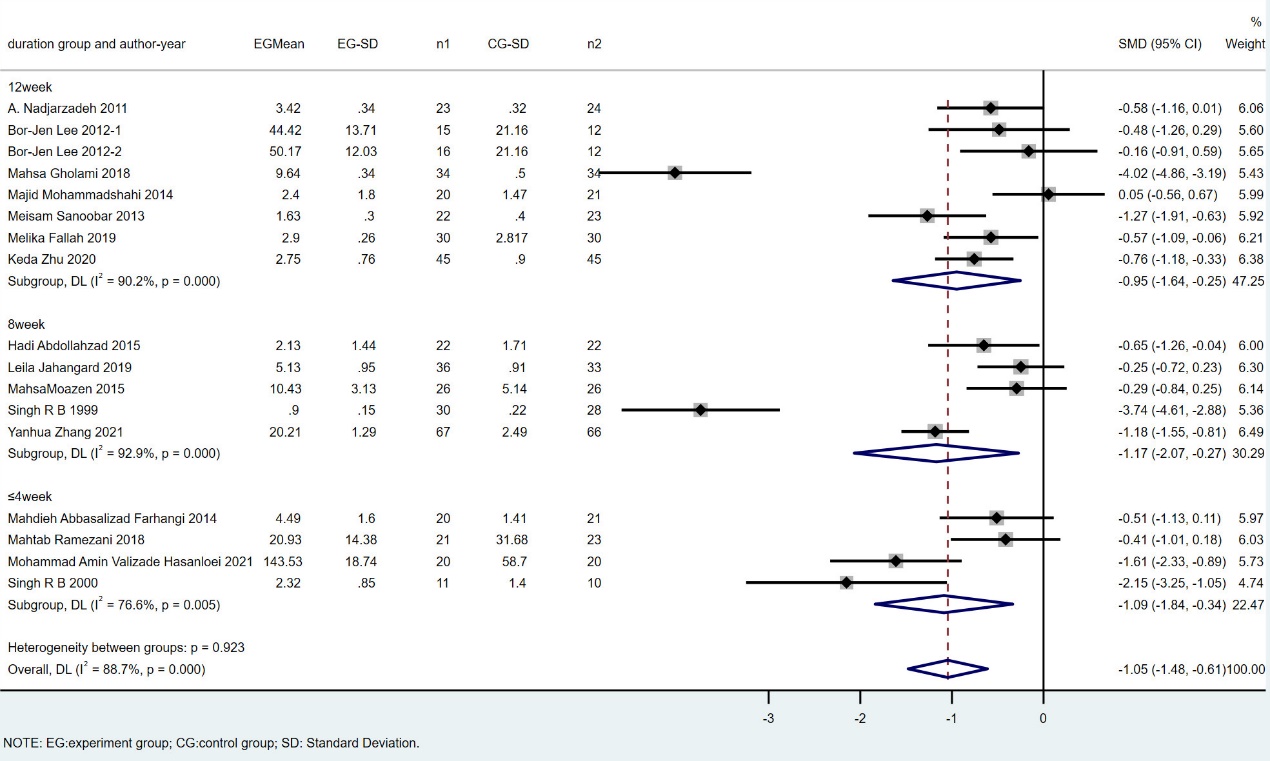


Supplementary Figure 1b: Meta-analysis presenting the effects of CoQ10 on MDA based on different duration subgroups. SMD: Stand Mean Difference; CI: Confidence Interval; EG: experiment group; CG: control group; SD: Standard Deviation.


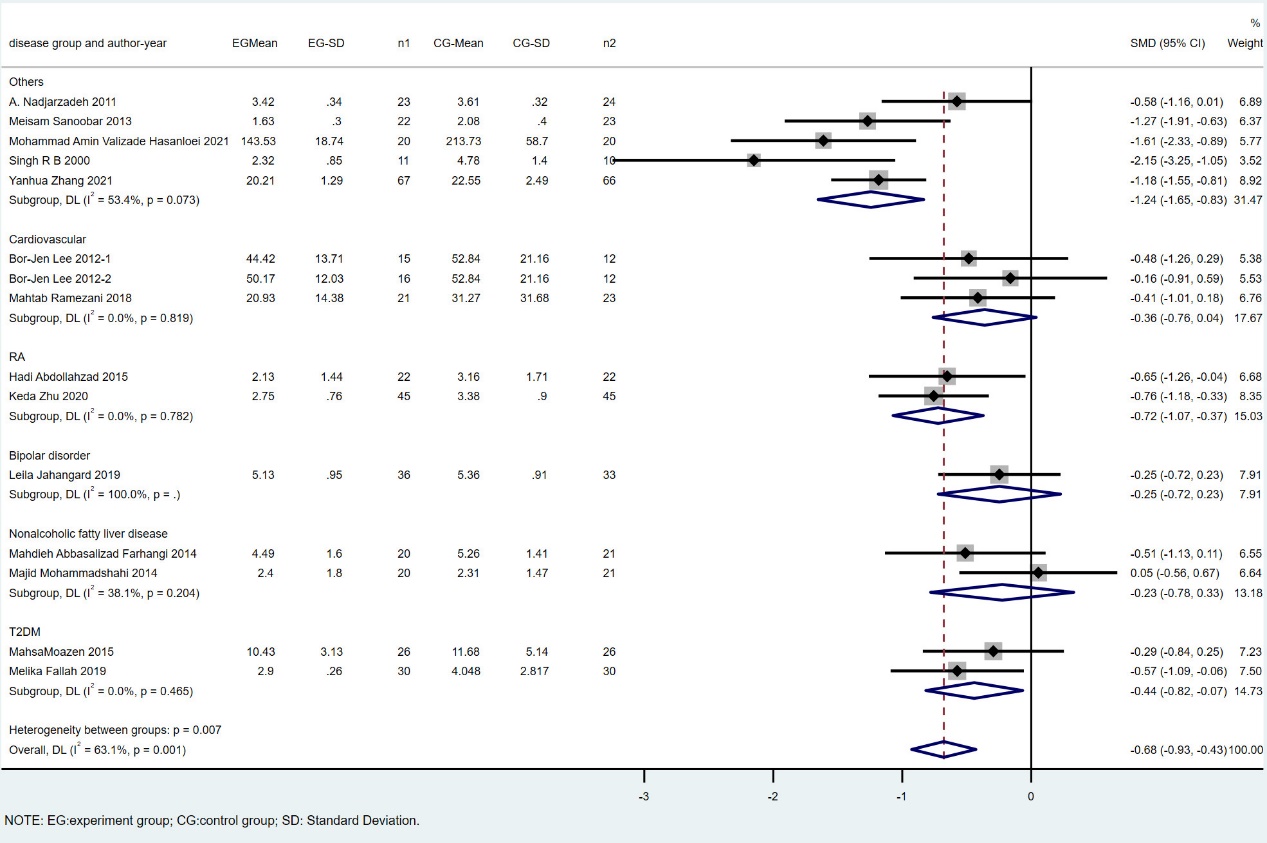


Supplementary Figure 1c: Meta-analysis presenting the effects of CoQ10 on MDA based on different diseases subgroups. SMD: Stand Mean Difference; CI: Confidence Interval; EG: experiment group; CG: control group; SD: Standard Deviation.

**TAC**


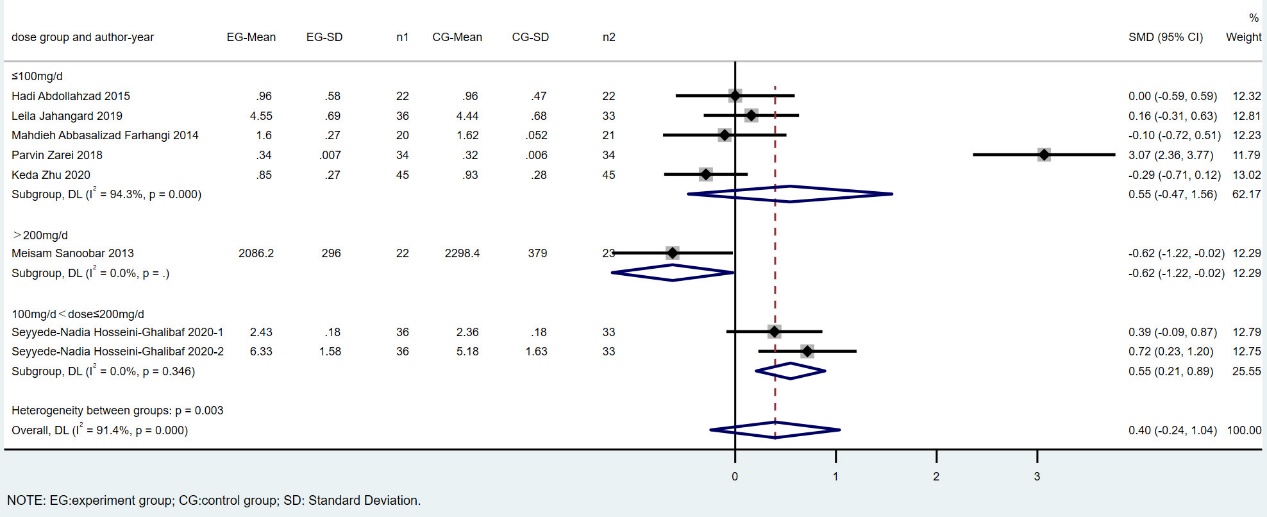


Supplementary Figure 2a: Meta-analysis presenting the effects of CoQ10 on TAC based on different dosages subgroups. SMD: Stand Mean Difference; CI: Confidence Interval; EG: experiment group; CG: control group; SD: Standard Deviation.


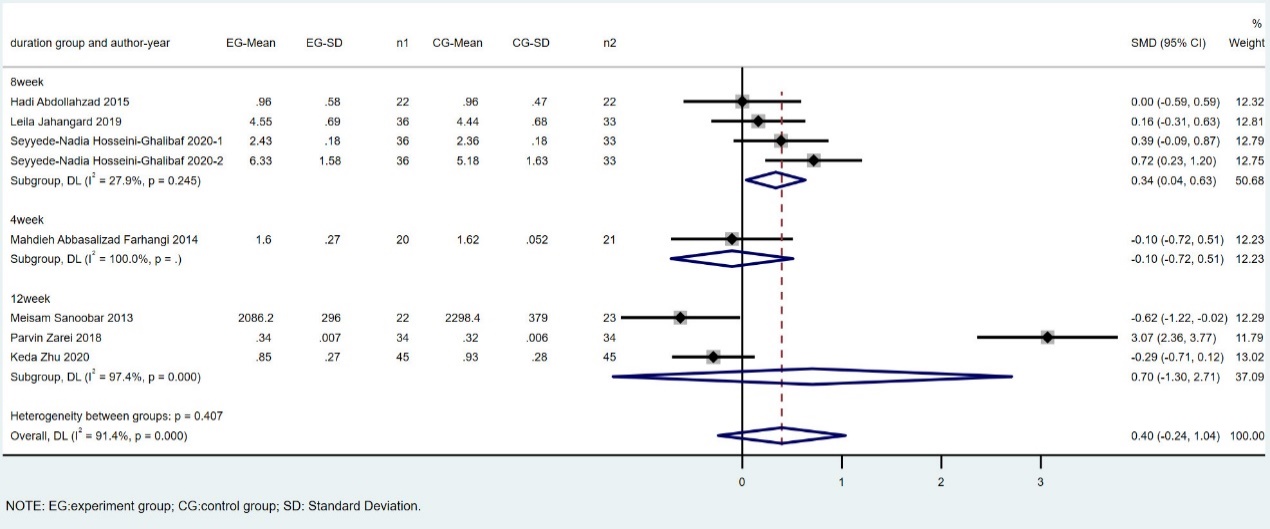


Supplementary Figure 2b: Meta-analysis presenting the effects of CoQ10 on TAC based on different duration subgroups. SMD: Stand Mean Difference; CI: Confidence Interval; EG: experiment group; CG: control group; SD: Standard Deviation.


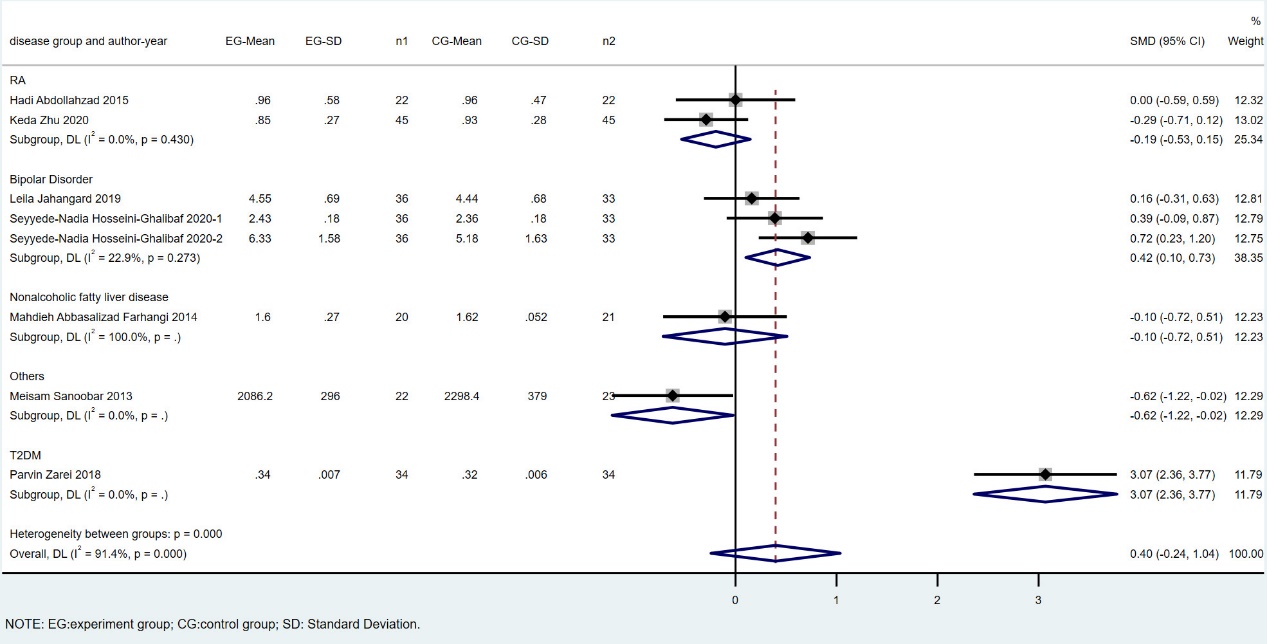


Supplementary Figure 2c: Meta-analysis presenting the effects of CoQ10 on TAC based on different diseases subgroups. SMD: Stand Mean Difference; CI: Confidence Interval; EG: experiment group; CG: control group; SD: Standard Deviation.

**CAT**


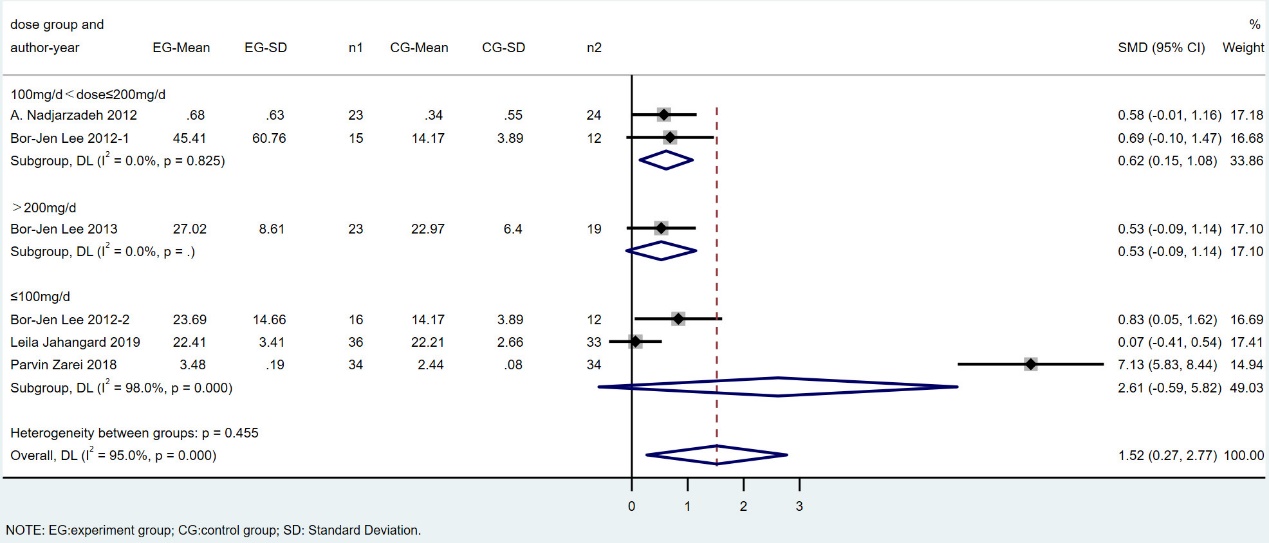


Supplementary Figure 3a: Meta-analysis presenting the effects of CoQ10 on CAT based on different dosages subgroups. SMD: Stand Mean Difference; CI: Confidence Interval; EG: experiment group; CG: control group; SD: Standard Deviation.


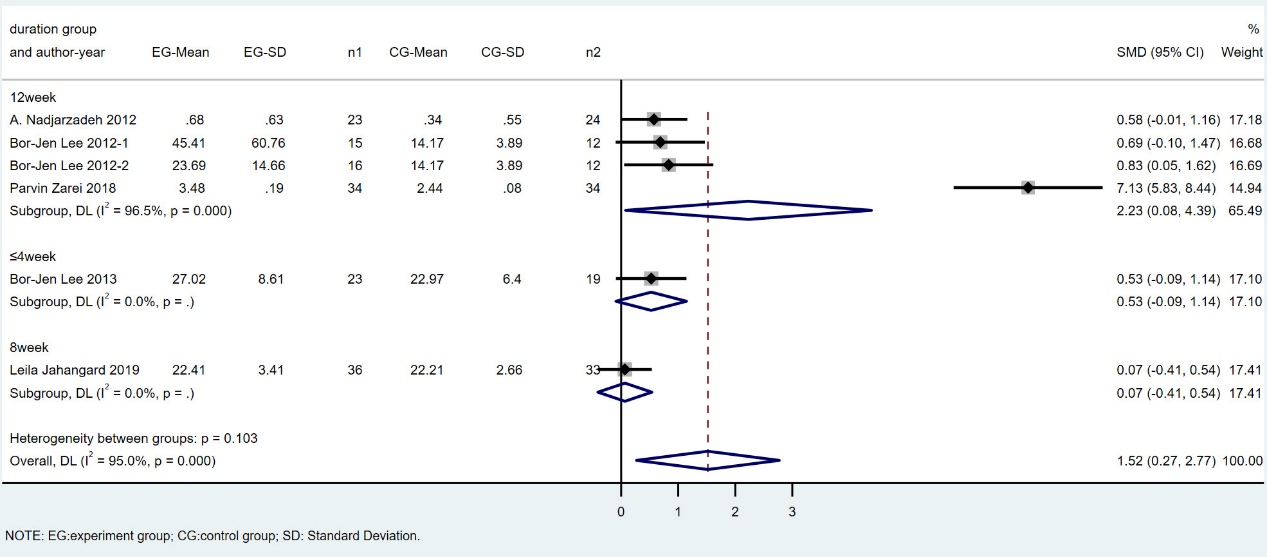


Supplementary Figure 3b: Meta-analysis presenting the effects of CoQ10 on CAT based on different duration subgroups. SMD: Stand Mean Difference; CI: Confidence Interval; EG: experiment group; CG: control group; SD: Standard Deviation.


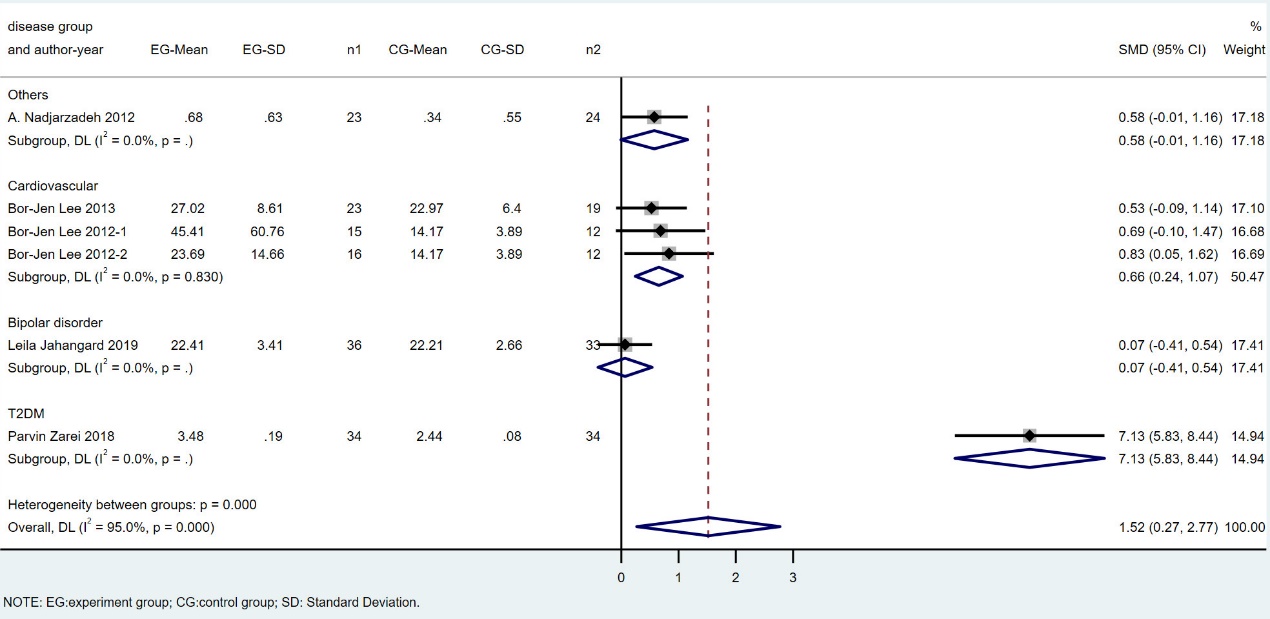


Supplementary Figure 3c: Meta-analysis presenting the effects of CoQ10 on CAT based on different diseases subgroups. SMD: Stand Mean Difference; CI: Confidence Interval; EG: experiment group; CG: control group; SD: Standard Deviation.

**GPx**


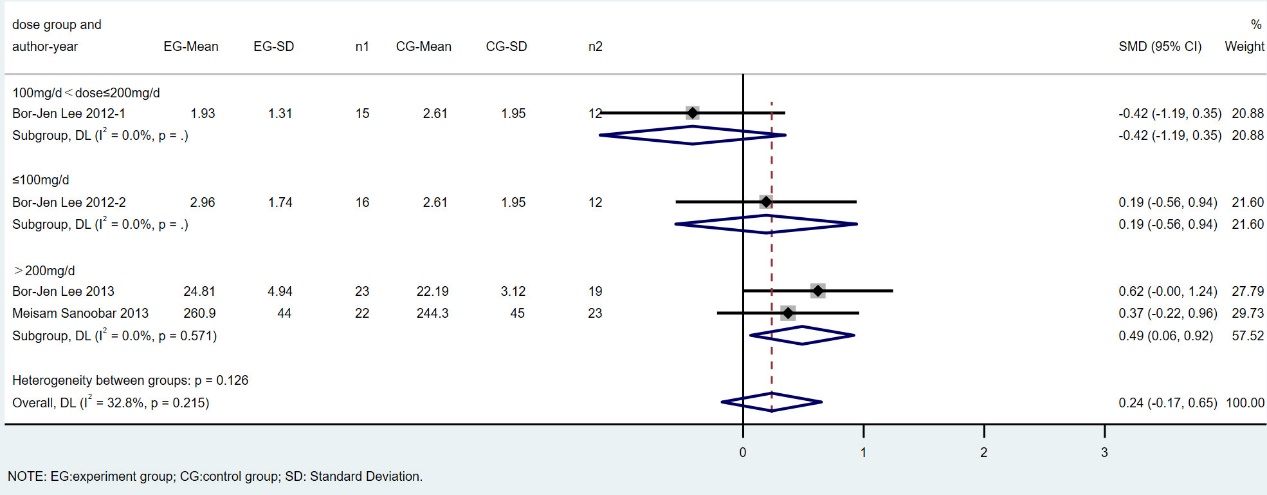


Supplementary Figure 4a: Meta-analysis presenting the effects of CoQ10 on GPx based on different dosages subgroups. SMD: Stand Mean Difference; CI: Confidence Interval; EG: experiment group; CG: control group; SD: Standard Deviation.


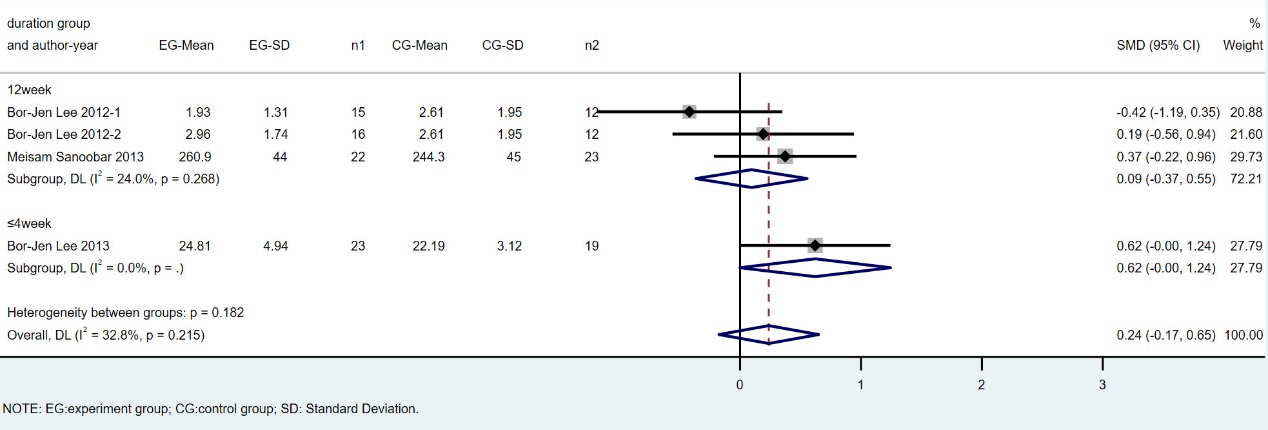


Supplementary Figure 4b: Meta-analysis presenting the effects of CoQ10 on GPx based on different duration subgroups. SMD: Stand Mean Difference; CI: Confidence Interval; EG: experiment group; CG: control group; SD: Standard Deviation.


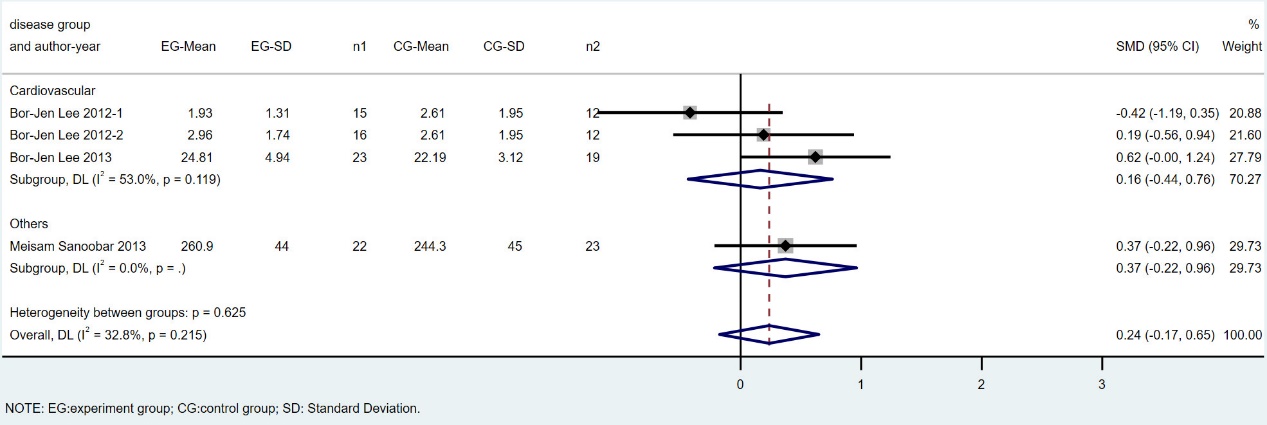


Supplementary Figure 4c: Meta-analysis presenting the effects of CoQ10 on GPx based on different diseases subgroups. SMD: Stand Mean Difference; CI: Confidence Interval; EG: experiment group; CG: control group; SD: Standard Deviation.

**Sensitivity analysis**

**MDA**

Supplementary Figure 5: Sensitivity analysis of the MDA effect of each primary study on the pooled results. CI: Confidence Interval.

**TAC**

Supplementary Figure 6: Sensitivity analysis of the TAC effect of each primary study on the pooled results. CI: Confidence Interval.

**CAT**

Supplementary Figure 7: Sensitivity analysis of the CAT effect of each primary study on the pooled results. CI: Confidence Interval.

**GPx**

Supplementary Figure 8: Sensitivity analysis of the GPx effect of each primary study on the pooled results. CI: Confidence Interval.
